# Development of a co-produced tool for monitoring and supporting the mental health of young people

**DOI:** 10.1192/bjo.2021.711

**Published:** 2021-06-18

**Authors:** Joy MacKeith, Anna Good, Sara Burns

**Affiliations:** Triangle

## Abstract

**Aims:**

The aims were to develop and validate a tool for monitoring and supporting the mental health of young people. Based on extensive experience of developing similar tools, the hypothesis was that a user-friendly tool could be produced with sound psychometric properties.

**Background:**

The Outcomes Star is a suite of collaboratively completed, strengths-based tools with the dual roles of both supporting and monitoring change. Service users are empowered through their active involvement in identifying their strengths and creating their care plan. Triangle, the creators of the Outcomes Star was approached by a number of organisations to develop a version of the Star for young people with mental health issues in early intervention services and also to support young people in managing a diagnosed mental illness.

**Method:**

Using a series of focus groups and an iterative process of refinement we gathered data from practitioners and service users on the domains in which they wish to create change, and the steps of the change process. A draft version of the new tool was piloted in two organisations by 67 workers and 177 young people over six months. The pilot data were analysed to assess the psychometric properties of My Mind Star (acceptability, skew, factor structure, internal consistency, item redundancy and responsiveness).

**Result:**

The resulting tool, My Mind Star consisted of seven domains: Feelings and emotions, Healthy lifestyle, Where you live, Friends and relationships, School, training and work, How you use your time and Self-esteem. Almost all young people and practitioners (94%) agreed that their completed Star was ‘a good summary of my life right now’ and that it gave a better idea of service users’ support needs. Psychometric analyses indicated a unidimensional structure with good internal consistency (α = .76) and no item redundancy. My Mind Star was responsive to change between the first and second readings, with medium and small-medium effect sizes.

**Conclusion:**

Initial findings suggest that My Mind Star has good psychometric properties and is perceived as acceptable and useful by young people and practitioners, Further research is planned to conduct a full validation of the psychometric properties of this Star including inter-rater reliability and predictive validity.

Financial sponsorship of the study: Action for Children

